# The Life-Cycle Costs of School Water, Sanitation and Hygiene Access in Kenyan Primary Schools

**DOI:** 10.3390/ijerph13070637

**Published:** 2016-06-27

**Authors:** Kelly T. Alexander, Alex Mwaki, Dorothy Adhiambo, Malaika Cheney-Coker, Richard Muga, Matthew C. Freeman

**Affiliations:** 1 CARE USA, 151 Ellis St. NE, Atlanta, GA 30307, USA; mCheneyCoker@care.org; 2 Department of Environmental Health, Emory University, 1518 Clifton Road NE, Atlanta, GA 30322, USA; matthew.freeman@emory.edu; 3 CARE Kenya, Mucai Road P.O. Box 43864-GPO, Nairobi 00100, Kenya; alex@care.or.ke (A.M.); dadhiambo4@gmail.com (D.A.); 4 Department of Health Sciences, Great Lakes University of Kisumu, P.O. Box 2224, Kisumu 40100, Kenya; drmuga@yahoo.com

**Keywords:** life-cycle cost, school, WASH, sustainability

## Abstract

Water, Sanitation and Hygiene (WASH) programs in schools can increase the health, dignity and comfort of students and teachers. Understanding the costs of WASH facilities and services in schools is one essential piece for policy makers to utilize when budgeting for schools and helping to make WASH programs more sustainable. In this study we collected data from NGO and government offices, local hardware shops and 89 rural primary schools across three Kenyan counties. Current expenditures on WASH, from school and external (NGO, government, parent) sources, averaged 1.83 USD per student per year. After reviewing current expenditures, estimated costs of operations and maintenance for bringing schools up to basic WASH standards, were calculated to be 3.03 USD per student per year. This includes recurrent costs, but not the cost of installing or setting up WASH infrastructure, which was 18,916 USD per school, for a school of 400 students (4.92 USD per student, per year). These findings demonstrate the need for increases in allocations to schools in Kenya, and stricter guidance on how money should be spent on WASH inputs to enable all schools to provide basic WASH for all students.

## 1. Introduction

According to the World Health Organization, the Millennium Development Goal (MDG) target for “improved” access to drinking water supply was met in 2010, but coverage fell well short of the target for access to “basic” sanitation [1]. In both cases, the indicators did not measure if these systems provided “safe and sustainable access” to households [2]. Unlike the MDGs, which only tracked household access, the post-2015 Sustainable Development Goals target provision of “universal access to basic drinking water, sanitation and hygiene” (WASH) for schools and health facilities [3]. Data on school coverage are scarce, but UNICEF estimates that in Sub-Saharan Africa in 2013, 54% of schools had adequate access to water and 53% had access to sanitation. An estimated 21% of schools in low-income countries have handwashing facilities [1]. No data exist specifically for schools, but estimates in low income settings are that fewer than 20% of incidents of contact with feces—either toilet use or diaper changes—were followed by handwashing events [4]. This lack of reliable access to safe and sustainable WASH infrastructure, in conjunction with related hygiene and sanitation behaviors, account for millions of deaths per year [5,6], as well as adverse impacts on pupil health and school attendance and enrollment [7,8,9].

UNICEF and the WHO have guidelines for minimum standards for school WASH, particularly focused on resource-poor settings [10,11]. Although contexts are diverse, according to the WHO, WASH in schools should at a minimum have: close and safe sanitation facilities (separated by gender when possible), handwashing facilities with soap or other cleansing agent, access to safe water for drinking, hygiene promotion for students, and a fenced school compound to encourage a clean environment [10]. UNICEF underlines the importance of practicing “key hygiene behaviors” in order to reduce the disease transmission within the school and within families and the broader community [11]. The minimum standards for the Government of Kenya, comparable to the Sphere standards [12] are five liters of water per student per day (for drinking and handwashing), at least one handwashing station per set of latrines, and a 25:1 student latrine ratio for girls (30:1 for boys with 1 meter of urinal wall for 50 boys) [13].

Even with appropriate provision of water supply and toilets, maintenance of infrastructure and sustainability of hygienic sanitation and water behaviors have been crucial school WASH challenges in low-income settings. In Kenya, 2.5 years after a handwashing and water treatment intervention, 36% of 55 schools had drinking water available, 16% of schools provided handwashing water, and only one school had soap [14]. Another study conducted in 100 urban and rural schools in Kenya found that nearly 50% of schools had no handwashing facilities, and nearly 50% had dirty, or poorly maintained toilets [15]. Soap was found to be missing in the majority of schools in a UNICEF pilot study in six countries, greatly limiting the ability of children to practice what they are learning in schools [16]. These examples demonstrate the challenge many schools face: WASH infrastructure, often established by NGOs or government partners, falls into disrepair, or cannot be used due to missing inputs. School infrastructure is more likely to last where there are “local champions” and engagement of the school and community prior to construction [17,18]. Schools with financial capacity and involvement of the school management committee—those who decide how the budgets are spent—are also more likely to have improved conditions for students [18,19]. Although there are inevitably additional factors that affect the availability and provision of water, soap and clean, private latrines, financial limitations are by and large the most consistent challenge schools face in maintaining their WASH services for students. However, most of the data we have on limited school budgets for WASH come from head-teacher or principal accounts, and not specific data on how money is allocated or spent.

One approach to improving sustainability of school WASH and address constraints associated with limited financial capacity is to improve the data available to policy makers on the full “life-cycle” costs of facilities. An approach to calculate these life-cycle costs for communities was developed by the IRC [20,21]. This approach assesses the costs for setting up, maintaining and sustaining water and sanitation systems [22]. This method did not initially include an approach for assessing life-cycle costs for school-based programs, however in 2013 IRC conducted WASHCost research in 117 schools in Bangladesh [23].

More generally, the Life Cycle Costs Approach (LCCA) is a methodology used for estimating future costs [24,25]. The LCCA was originally used by the U.S. military, and frequently in the industrial sector to estimate direct and indirect costs of items and activities from inception to distribution. Costs are calculated in a number of ways, most often through reviewing past and recent costs, looking at “analogous” costs, or those that may be similar in nature (though from a different region or industry), and making an educated estimate [24,25]. Within the WASH sector, LCCA is used to aggregate the costs of installing, maintaining and sustaining WASH infrastructure. There are six main types of costs which make up the life-cycle costs of WASH: (1) capital hardware and software; (2) capital maintenance; (3) cost of capital; (4) operating and minor maintenance; (5) direct support and (6) indirect support [26]. Capital hardware costs are the costs of establishing infrastructure at the school and, as mentioned above, capital software is the training of teachers, students and parent teacher association (PTA) members. Operation and minor maintenance costs may include hinges or locks on latrine doors—to provide properly functioning doors and privacy—supplies for cleaning latrines, water treatment for making water safe to drink, soap for handwashing, or taps for water dispensers. Capital maintenance costs are larger costs for maintaining or repairing WASH infrastructure at the school. Direct and Indirect support are costs of government monitoring and advocacy, respectively.

The smaller operational items, or recurrent costs, are those that are often most important to student health and comfort. Students, and girls in particular, have expressed the need for water, soap and clean latrines at school [27]. Girls have reported that they will often go home, or skip school, when they need to manage their period if the facilities available are inadequate [28,29,30,31,32]. This challenges the basic rights of girls’ to have the same chance as boys to excel in school. It is difficult to perform at the same level of their male peers if females need to miss class for a few hours or days each month [33,34,35]. Latrine cleanliness affects student use, and may be more important than the sheer number of latrines–evidence supporting the importance of maintenance and recurrent costs over simply building infrastructure [9,36]. One study found that use of latrines without handwashing and soap facilities, was linked to increased fecal matter on hands [37], supporting previous work that handwashing with water alone is not as effective at reducing risk of disease as handwashing with soap [38,39]. Though findings have been mixed, sub-optimal school WASH has been linked to girls’ attendance, soil-transmitted helminth infection, diarrhea and respiratory infection [7,8,40,41,42,43].

Understanding the life-cycle costs of WASH facilities and services in schools would support program planners and policy makers to better allocate funds for school-based WASH programs. For example, allocation towards maintenance and repair of facilities before the facilities become unusable, might prove more cost effective than repeated capital investments in new infrastructure. Here we report on a study to assess the life-cycle costs of school WASH in Kenya. The study was nested within Sustaining and Scaling School WASH plus Community Impact (SWASH+), an applied research project with the objective to generate high quality data to support government stakeholders to make evidence-based decisions regarding school WASH. The purpose of this study was to answer the following research questions: What are the current expenditures related to school WASH in rural Kenya? What is the estimated cost of providing schools in rural Kenya with WASH that meets minimum standards?

## 2. Materials and Methods

Data collection took place in three counties of Kenya: Kisumu, located in the Western part of the country abutting Lake Victoria; Nyeri, located relatively near the capital of the capital city Nairobi in the mountainous central plateau; and Kilifi, a predominantly Muslim area located on the Indian Ocean. These three counties were selected by CARE Kenya, with the input of the Government of Kenya, (GoK) due to the diversity of geography and population. We utilized six of the seven domains developed by the WASHCost project to assess life-cycle costs (Table 1):

For school WASH costs, as shown above, capital software was split from capital hardware since teaching and training about hygiene is quite different than purchasing or installing latrines. Cost of capital is a domain regarding interest payments on loans taken out by the school—and this was not applicable to the Kenyan school context, since none of the schools reported applying for loans. 

Eligible schools included all listed government primary schools in Kisumu, Nyeri and Kilifi. Thirty schools were randomly selected from each county school list using a random number generator. Data were collected between May and July 2014 by two staff members with extensive work experience in the school WASH sector in Kenya and entered into Excel (Microsoft, Redmond, WA, USA) using pre-defined entry sheets to divide costs between “actual” expenditures and “estimated” costs. Data were double-entered and checked for consistency in SAS 9.3 (SAS Institute Inc., Cary, NC, USA).

### Analysis

We quantified the financial source of WASH expenditures in the school, as well as categorized the domain (capital hardware, operation and maintenance, capital software, etc.) and the type of item (latrines, soap, sanitary pads) paid for by that source (government, NGO, parents or the school). We report the expenditures “per capita”, using the amount spent on an item divided by the number of students the item was meant to serve. Data for current WASH expenditures came from principal reports, NGO and government interviews.

We then calculated the average cost of each WASH item by comparing data from school principal reports (current expenditures), principal estimates for repairs, price data from local shops, and what we considered to be the gold standard, NGO and government office financial reports. Each WASH item was assigned an average cost, and average “lifespan”, based on data collected. For consumables and maintenance costs, we derived estimates from school visit data on the length of time, for example, to use x number of bars of soap for x number of students. For the frequency of repairs, such as latrine door latches, we averaged how often schools needed to make these repairs. Basic water hardware included either a borehole or rainwater catchment, including gutters and four storage tanks, as some schools were not in a geographic area where drilling a borehole was feasible. For a school of 400 students, 16 latrine pits were calculated as meeting the required GoK standards [13] for basic school WASH. Water vessels, including four large ones for handwashing and four smaller ones for drinking, were calculated using the GoK guidelines (checked for consistency with the Sphere guidelines) for minimum water allowance at school [12]. We went beyond the minimum standards, as described by WHO and UNICEF, to include sanitary pads, locks for latrine doors and water in girls’ latrines (see Supplementary Table S1 on basic school WASH for more details). Item costs were divided into life-cycle cost domains in order to calculate a cost per domain. We calculated water and sanitation capital hardware as lasting 10 years, since this is two Government of Kenya budgeting cycles, and a reasonable estimate considering an average of nearly 50% of school WASH infrastructure is in need of replacement or major repairs [44]. Drinking water and hygiene infrastructure items were calculated as lasting only three years, since these items need to be replaced more regularly. Capital software and operation and maintenance costs were calculated annually. 

The study was not subject to ethics approval, as it is not classified as human subject research. We had written approval from the Government of Kenya to visit schools and requested permission and consent from teachers to visit the school and ask them about WASH infrastructure.

## 3. Results

Data were collected from 89 schools, six NGOs, three local government offices and seven hardware shops. The principal at one school did not consent to participate. Data was verified by multiple data sources to ensure higher accuracy, however the estimated costs of items made by school informants were similar to the costs collected from shops or reported by NGOs and government officials, so little adjustment to estimates was needed. 

### 3.1. Current Expenditures

School WASH expenditures are made through a combination of NGOs, government offices (in this case grants from the Constituency Development Fund [CDF], government resources devolved from the federal to local level), parents and school budgets, allocated to them three times a year by the GoK. In Table 2, the second column lists the breakdown of all reported WASH expenditures, according to the source of the item. Columns 3, 4 and 5 show the breakdown of expenditures of WASH—by the percentage that source contributed to that category of expenditure. The majority of all funds spent by all sources of support was on sanitation (64%), followed by water (35%), with 1% spent on hygiene. NGOs were the source of 39% of all WASH expenditures at the school. Specifically, 77% of all hygiene costs were paid for by NGOs—covering the costs of handwashing vessels, soap and sanitary pads. CDF funded 12% of sanitation costs, meaning that for a few schools, latrines were built using CDF funds, and for others, there was no CDF contribution. Schools apply for CDF grants, usually with the support of the local government, to receive these funds*.* The parent teacher associations (PTAs) covered 20% of costs for water and sanitation (and to a lesser extent hygiene), demonstrating that parents greatly contribute to WASH costs—over half of NGO contributions and more than school expenditures. PTA expenditures mainly covered repairs to water sources and latrines and salary for the school security guard. Security guards were reported by schools to be part of their WASH costs due to watching over the water point and latrines.

Capital hardware, or WASH infrastructure such as latrines, handwashing stations and water tanks, were the greatest costs and mainly paid for by NGOs. Sixty-five percent of all water costs, an average of 2825 USD (251,135 KES) per school were covered by NGOs (Table 2). Additionally, 36% of all sanitation costs, an average of 10,400 USD (932,337 KES) and 77% of all hygiene costs, an average of 23 USD (2070 KES) per school were paid for by NGOs. WASH expenditures averaged nearly 34,000 USD per school—this included any (functional or non-functional) WASH infrastructure for which the teachers could recall the cost.

Software expenditures were measured by the time teachers spent monthly instructing on handwashing and latrine use, or buying supplies for the WASH program. Teachers spent anywhere from 0–100 min a month (0–25 min per week) talking about WASH-related practices, and spent 0–4 ho each month purchasing supplies. Twenty to 30 students spent on average 20 min cleaning latrines and filling water containers each day. 

From the government-allocated budget, schools, with an average of 511 students, spent on average 76 USD (6780 KES) per year on minor repairs for their water supply, and 55 USD (4889 KES) per year on recurrent costs for water (such as water treatment and security guard salaries). For sanitation, costs were 80 USD (7136 KES) per year for minor repairs, and nearly 30 USD (2667 KES) for recurrent costs per school per year. Only eight (9%) schools reported they used budget for hygiene (as mentioned above 77% of hygiene costs were covered by NGOs). Average hygiene expenditures, 1.85 USD for minor repairs and 1.60 USD for recurrent costs, are not representative figures since a few schools spent a lot more than two dollars each year, and many schools spent nothing at all. Total WASH expenditures, on average across the 89 schools, was 1.83 USD (163 KES) per student or 732 USD (65,299 KES) per school from all sources, each year.

Expenditures on direct and indirect support for school WASH were not easily interpreted by our tools, and thus they were not included in our life-cycle cost calculation. Direct support includes time government staff and teachers spent on monitoring school WASH facilities—and this varied greatly from 0 to 30 min a day (per school). Indirect support, or time spent on advocacy and policy-making for school WASH, was also not easily interpretable as this varied from 1–2 days per year to more than 20 days per year (e.g., if an NGO staff spent time advocating the GoK for improved school WASH conditions). No estimates were given on the amount of time teachers spend monitoring WASH facilities and checking to see that everything is in working order. 

Expenditure data was split by county to explore if expenditures were comparable, using three items per domain (Table 3). For some items, such as soap for handwashing, there were large differences across counties, with nearly 50 USD spent in Kisumu county and 3 USD and 7 USD spent in the other counties (there were more donors purchasing soap for the 30 sample schools in Kisumu). Meanwhile door repair expenditures were within 7 USD across the three counties and adding together expenditures from water, sanitation and hygiene domains, total expenditures were within 60 USD.

### 3.2. Estimated Costs

Thirty-four percent of schools reported needing repairs to their water facilities, such as cementing around a borehole, or replacing tubes, pipes or gutters. All schools visited needed repairs done to their latrine facilities. Examples of needed repairs included resurfacing floors or walls, improving drainage, emptying latrines, or replacing doors or vent pipes. Hygiene facilities, such as handwashing vessels, were all either in need of an upgrade, (new containers or taps that did not leak), or schools had no handwashing facilities at all. 

We calculated the life-cycle costs for establishing and maintaining a school WASH program for 10 years as 31,786 USD (2,825,422 KES), or 3178 USD (282,542 KES) per school per year. In a school of 400 students, that amounts to about 8 USD per student, per year. This figure includes building infrastructure (capital hardware), using the rate of 18,590 USD (rainwater system with VIP latrines), 326 USD times 3.3 years (Table 4) and 1212 USD times 10 years (Table 5).

The cost for each school, every three years for hygiene infrastructure, is estimated at 326 USD total, or 108 USD per year (Table 4). Yearly capacity-building on the use of WASH infrastructure and WASH monitoring for teachers, PTAs and government officials, (capital software, direct support) is expected to be 334 USD per year. This price can be adjusted down if trainings do not occur on a yearly basis for each school. Maintenance costs for minor repairs include items such as locks for latrine doors, taps for handwashing stations and realignment of gutters. Costs of major repairs were 0.65 USD (60 KES) per student per year (Table 5). The total cost of minor repairs was 0.33 USD (30 KES) per student per year, or 134 USD per school per year (for a school of 400 children). Operations costs, which included latrine cleaning supplies, soap for handwashing, water treatment and sanitary pads, among other items, totaled 2.02 USD per student or 806 USD per school per year. This included half of the salary of a security guard, with the other half paid for directly by PTA. Overall, operations and maintenance costs, or recurrent costs, for a school of 400 students is estimated at 1212 USD per year, or 3.03 USD per student per year (Table 5). 

## 4. Discussion

Sustainability of school WASH is a critical challenge in low resource environments. Understanding the life-cycle costs for WASH, specifically for school WASH, will support policy makers and implementers in appropriately allocating sufficient funds to support healthy school environments. In order to answer our first research question, we asked schools about their current WASH expenditures. This was found to be on average, 1.83 USD (163 KES) per student per year. There were large discrepancies between schools, particularly for hygiene, considered to be one of the most cost-effective public health interventions [45]. We also saw differences in expenditures across the three counties on a *per item* basis, which is not surprising considering some schools had outside support or prioritized WASH, while others had little inputs. These discrepancies—with only some schools meeting minimum standards for WASH—is consistent with the overall picture of WASH in Schools in Kenya [46]. A study conducted by UNICEF in Kenya found that less than 25% of schools met the student latrine ratios, less than 50% had safe water and 9.3% of schools had basic hygiene facilities [46]. It was also reported that the Government of Kenya focused on latrine building over supporting items needed for behavior change [46]. Increasing investments in operation and maintenance would, in the long term, reduce the need for reinvestment in broken water systems and unusable, dirty or unsafe toilets. 

The second research question investigated the costs of supporting schools to meet minimum standards for a healthy school environment. We found that the recurrent costs are approximately 3.03 USD per student per year–which is quite a bit higher than the results of the costing study conducted in Bangladesh that found schools needed 1.40 USD per student per year [23]. One reason for our higher estimates is that the “minimum standards” we set for estimating costs for schools includes more considerations than the WHO or UNICEF guidelines. For example we included sanitary pads, toilet paper, a water container in the girls’ latrine, and cleaning supplies for latrines. These items have been found to be critical for a healthy school environment for students. We also considered locks on latrines as essential for student dignity and privacy. Our recurrent costs can be lowered to 2.22 USD by taking out toilet paper (many students in Kenya have never used toilet paper at school or home) [47], or down to 1.72 USD per student by removing costs of a school security guard (see the budget worksheet, Supplementary Table S2 in excel 2). Overall, however, we found that it is approximately 8 USD per year per student (for 10 years) to initiate and maintain a WASH program at a new school—and the study in Bangladesh found a similar amount of 10 USD per student for building WASH infrastructure. For schools with existing WASH infrastructure: sufficient latrines, handwashing facilities and a water source that meets the needs of the school population, the amount of 3.03 USD per student per year should be sufficient for maintaining the minimum standard for basic school WASH.

The Government of Kenya revised the allocations for schools in the 2014/2015 financial year from 11.43 USD (1020 KES) to 15.92 USD (1420 KES), per student per year. Of this amount, 2.20 USD (225 KES) per student per year, up from 1.34 USD (137 KES) is *available* for costs that may include WASH items. This does not mean that 2.20 USD is spent on WASH specifically, but instead could be spent on important classroom repairs. Allocations to schools are made per vote head (budget-line) such as Repairs, Maintenance and Infrastructure: 1.12 USD (100 KES), and Electricity, Water and Conservancy: 0.67 USD (60 KES). The GoK approved a new vote head called Environment and Sanitation: 0.56 USD (50 KES) and added “Sanitary Towels” 0.17 USD (15 KES) to the Contingency description. These small changes demonstrate the increased efforts on the part of the GoK to recognize school WASH and its important role within the school environment. According to our data, yearly recurrent costs are 3.03 USD (270 KES) per student, a shortfall of at least 0.40 USD (45 KES) per student per year. Schools often do not, or are unable to, prioritize items required to maintain their WASH program [19]. For this reason, we suggest a WASH-specific budget-line for schools, with supporting recommendations on how money should be used to improve the environment for students and teachers.

A strength of this study was that we visited 89 schools in three diverse counties of Kenya. A limitation was that we only collected data from three of the forty-seven counties in Kenya, so although schools were randomized, counties were selected purposively, as they were seen to be diverse from each other. Another challenge was that in each county, many schools had very few WASH expenditures, while others prioritized WASH and had much higher expenditures, meaning that calculated average expenditures were not always an accurate reflection of the “average” school. Life-cycle costs were calculated from these average expenditures, along with limited data from government and NGO offices. Although very few of these were sampled, figures were nearly identical, so further data collection would likely not have changed the cost estimates.

The increased budget allocations in 2015 demonstrate that the GoK is paying attention to the evidence of school WASH research and responding to the advocacy work that has pushed for this increased school WASH budget [48]. Money spent on establishing infrastructure, like water sources or latrines, is often contributed by international NGOs, limiting the sustainability of school WASH programs in the longer term, particularly for schools that have not received external support. Sufficient funding for minor maintenance and operations costs would support schools to maintain the infrastructure they have. Financing options for new schools, or those with lower populations, would support schools that require additional (not solely capitation-based) budgets, in order to afford adequate school WASH inputs.

## 5. Conclusions

We calculated the current expenditures for school WASH in three counties in Kenya and estimated life-cycle costs required for schools to meet the minimum standards. Current annual expenditures fall within the limits of current GoK allocations—however this does not mean schools have, or purchase what they need for students since many resources are coming from outside the school. There needs to be an increased allocation for schools, so that all schools can maintain minimum WASH standards for students. We also recommend creating a specific budget-line for WASH items, and developing stricter guidance to accompany the WASH budget-line. To our knowledge, this is the first study published on current expenditures on school WASH and the first to calculate the actual expenditures and life-cycle costs of school WASH. Although this study was originally undertaken to inform GoK policy on school WASH budgets, these findings could inform policy discussions on school WASH in other low resource settings. The results of this study have led to an ongoing discussion within the Ministry of Education, Science and Technology in Kenya, to increase allocations to schools. 

## Figures and Tables

**Table 1:** Life-cycle cost domains and their descriptions in the context of school WASH, Kenya, 2014.

Life-Cycle Cost Domain	Description and Examples of Domain in School WASH Context
Capital hardware	Capital investment in fixed assets. For sanitation: excavation, lining, slabs, superstructure. For water: borehole, rainwater harvesting system, storage containers. For hygiene: handwashing stations, vessels.
Capital software	Training teachers and students on proper latrine use, water handling and treatment/filtration; handwashing techniques and key times, local monitoring.
Operation and minor maintenance	Costs of time spent cleaning latrines, or collecting water. Latrine cleaning and water treatment materials, hygiene supplies. Minor repairs.
Capital maintenance	Costs of rehabilitating latrines, fixing drainage, latrine emptying; rehabilitating water point, replacing handwashing vessels or fixing handwashing stations.
Direct support	Costs of monitoring school WASH systems by government officials.
Indirect support	Costs of macro-level support including advocacy and policy-making.

**Table 2:** Breakdown of reported source of funding for each WASH component and expenditure of water, sanitation and hygiene by that source—data from 89 schools across three counties in Kenya, 2014.

Source	WASH % Total Funding	Water % Total Funding (% Water Funding)	Sanitation % Total Funding (% Sanitation Funding)	Hygiene% Total Funding (% Hygiene Funding)
Donor/NGO	39%	14% (65%)	24% (36%)	~1% (77%)
School budgets	14%	6% (14%)	8% (32%)	<1% (15%)
Parent teacher association (PTA)	20%	11% (20%)	8% (20%)	~1% (5%)
Constituency Development Fund (CDF)	27%	4% (1%)	12% (12%)	<1% (3%)
Total support from all sources:	100%	35% (100%)	64% (100%)	~1% (100%)

**Table 3:** Average expenditures of schools for select items per WASH domain, by county, Kenya 2014.

Item	County USD (KES)		
	Kilifi	Nyeri	Kisumu
**Water**			
Security guard	666 (59,192)	598 (53,192)	548 (48,716)
Gutter repair	79 (6988)	78 (6933)	138 (12,309)
Tap repair	39 (3445)	44 (3894)	24 (2130)
**Sanitation**			
Brooms	13.5 (1200)	30 (2700)	14 (1281)
Disinfectant	76 (6788)	62 (5500)	35 (3083)
Door repair	73 (6500)	80 (7100)	79 (7029)
**Hygiene**			
Tap repair for handwashing	11 (1000)	17 (1500)	19 (1700)
Soap for handwashing	7 (585)	3 (240)	48 (4256)
TOTAL	964 (85,698)	912 (81,059)	906 (80,504)

**Table 4:** Setting up a school WASH program: Capital Hardware costs, Kenya 2014.

Item	Unit Cost of Item	Average Cost per School per Year	Average Cost per Student per Year
Total cost, divided by ten years	USD (KES)	USD (KES)	USD (KES)
Borehole	9529 (850,000)	953 (85,000)	2.38 (212)
Two 10,000 L water tanks	2690 (240,000)	369 (24,000)	0.67 (60)
Two 25,000 L water tanks	6726 (600,000)	673 (60,000)	1.68 (150)
Gutters for collecting rain water	202 (18,000)	20 (1800)	0.05 (4.5)
VIP latrine: 4 doors	8965 (800,000)	897 (80,000)	2.24 (200)
Sub-total borehole and VIP	18,500 (1,650,000)	1850 (165,000)	4.62 (412.50)
Sub-total rainwater system and VIP	18,590 (1,658,000)	1859 (165,800)	4.65 (414.50)
Total cost, divided by three years			
Handwashing vessels	224 (20,000)	75 (6667)	0.19 (16.7)
Drinking water vessels	90 (8000)	30 (2667)	0.07 (6.7)
Water vessel for menstrual hygiene	12 (1000)	4 (333)	0.01 (0.8)
Sub-total	326 (29,000)	108 (9667)	0.27 (24.2)
TOTAL (higher cost of rainwater system)	18,916 (1,681,422)	1967 (175,467)	4.92 (439)

**Table 5:** Maintenance and recurrent costs per year for schools in Kenya, 2014.

Item	Average Cost per School per Year	Average Cost per Student per Year
*Maintenance costs (major repairs)*	USD (KES)	USD (KES)
General repairs to water hardware	45 (4000)	0.11 (10)
General repairs to sanitation hardware	54 (4800)	0.13 (12)
General repairs to handwashing hardware	31 (2800)	0.07 (7)
Maintenance of source	56 (5000)	0.14 (12.5)
Latrine pit emptying (1 pit per year)	38 (3400)	0.09 (8.5)
Cleaning of storage tanks	45 (4000)	0.11 (10)
Sub-total	260 (24,000)	0.65 (60)
*Maintenance costs (minor repairs)*		
Taps, pipers or gutters	45 (4000)	0.11 (10)
Latrines (locks, hinges, vent pipes, doors)	60 (5400)	0.15 (13.5)
Buckets and brooms	20 (1800)	0.05 (4.5)
Handwashing taps	9 (800)	0.02 (2)
Sub-total	134 (11,911)	0.33 (30)
*Recurrent costs*		
Water treatment	40 (3600)	0.10 (9)
Security guard	202 (18,000)	0.50 (45)
Detergent and disinfectant	121 (10,800)	0.30 (27)
Soap for handwashing	60 (5400)	0.15 (13.5)
Sanitary pads	60 (5400)	0.15 (13.5)
Toilet paper	325 (29,000)	0.81 (72)
Sub-total	806 (72,000)	2.02 (180)
TOTAL	1212 (108,000)	3.03 (270)

## Supplementary Materials

The following are available online at www.mdpi.com/1660-4601/13/7/637/s1, www.mdpi.com/1660-4601/13/7/637/s2, Table S1: Basic standards for school WASH, Table S2: A budget worksheet, shared with the Government of Kenya.

## Abbreviations

The following abbreviations are used in this manuscript:

WASH	Water, sanitation and hygiene
MDGs	Millennium Development Goals
CDF	Constituency Development Fund
GoK	Government of Kenya
NGO	Non-governmental organization
USD	United States Dollar
KES	Kenyan Shillings
SDG	Sustainable Development Goals
PTA	Parents Teachers Association
VIP	Ventilated Improved Pit latrine
DOAJ	Directory of open access journals
TLA	Three letter acronym
LD	linear dichroism
