# Supplementary material for: The Life-Cycle Costs of School Water, Sanitation and Hygiene Access in Kenyan Primary Schools
Source: Int J Environ Res Public Health. 2016 Jun 27;13(7):637. doi: 10.3390/ijerph13070637 (PMC4962178; doi:10.3390/ijerph13070637)
Supplement: Supplementary file 1 [file ijerph-13-00637-s001.pdf]

Supplementary Materials: The Life-Cycle Costs of School Water, Sanitation and Hygiene Access in Kenyan Primary Schools

Kelly T. Alexander, Alex Mwaki, Dorothy Adhiambo, Malaika Cheney-Coker, Richard Muga and Matthew C. Freeman

Table S1. Basic standards for school WASH [1,2].

Item	Description
Sufficient latrines	The Government of Kenya states that the ideal student:latrine ratio is 25:1 for girls and 30:1 for boys, with a urinal available.
Separate latrines for boys and girls	Boys and girls do not share the same facilities.
Privacy in latrines	Locks on latrine doors; latrines without large holes in walls or doors.
Clean latrines	Latrines should be clean enough (including non-offensive smell) so that students feel comfortable using them.
Handwashing water with soap	Handwashing water and soap provided to students every day and all day.
Clean, safe drinking water	Water that is treated or filtered and available to students every day and all day.
Hygiene education	All students taught about handwashing, and principles around latrine use and safe water. Students educated on menstruation.
Sanitary pads	Sanitary pads available for menstruating girls in case of emergency.
Anal-cleansing	Toilet paper or extra water containers and cups, or other anal-cleansing needs according to cultural practices.

References

1. Mooijman, A. *Water, Sanitation and Hygiene (WASH) in Schools*; UNICEF: New York, NY, USA, 2012.
2. WHO. *Water, Sanitation and Hygiene Standards in Low-Cost Settings*; Adams, J., Bartram J., Chartier, Y., Sims, J., Eds.; WHO Press, World Health Organization: Geneva, Switzerland, 2009.

© 2016 by the authors; licensee MDPI, Basel, Switzerland. This article is an open access article distributed under the terms and conditions of the Creative Commons by Attribution (CC-BY) license (http://creativecommons.org/licenses/by/4.0/).
